# A Robust Parameter Estimation Method for Estimating Disease Burden of Respiratory Viruses

**DOI:** 10.1371/journal.pone.0090126

**Published:** 2014-03-20

**Authors:** King Pan Chan, Chit Ming Wong, Susan S. S. Chiu, Kwok Hung Chan, Xi Ling Wang, Eunice L. Y. Chan, J. S. Malik Peiris, Lin Yang

**Affiliations:** 1 School of Publish Health, The University of Hong Kong, Hong Kong Special Administrative Region, China; 2 Department of Paediatrics and Adolescent Medicine, The University of Hong Kong, Hong Kong Special Administrative Region, China; 3 Department of Microbiology, The University of Hong Kong, Hong Kong Special Administrative Region, China; 4 HKU - Pasteur Research Centre, Hong Kong Special Administrative Region, China; 5 Squina International Centre for Infection Control, School of Nursing, The Hong Kong Polytechnic University, Hong Kong Special Administrative Region, China; Harvard School of Public Health, United States of America

## Abstract

**Background:**

Poisson model has been widely applied to estimate the disease burden of influenza, but there has been little success in providing reliable estimates for other respiratory viruses.

**Methods:**

We compared the estimates of excess hospitalization rates derived from the Poisson models with different combinations of inference methods and virus proxies respectively, with the aim to determine the optimal modeling approach. These models were validated by comparing the estimates of excess hospitalization attributable to respiratory viruses with the observed rates of laboratory confirmed paediatric hospitalization for acute respiratory infections obtained from a population based study.

**Results:**

The Bayesian inference method generally outperformed the classical likelihood estimation, particularly for RSV and parainfluenza, in terms of providing estimates closer to the observed hospitalization rates. Compared to the other proxy variables, age-specific positive counts provided better estimates for influenza, RSV and parainfluenza, regardless of inference methods. The Bayesian inference combined with age-specific positive counts also provided valid and reliable estimates for excess hospitalization associated with multiple respiratory viruses in both the 2009 H1N1 pandemic and interpandemic period.

**Conclusions:**

Poisson models using the Bayesian inference method and virus proxies of age-specific positive counts should be considered in disease burden studies on multiple respiratory viruses.

## Introduction

Acute respiratory infections accounted for 11–22% of global deaths of children under five, with a significant proportion caused by respiratory viruses [1]. However, obtaining reliable population based estimates for disease burden of respiratory viruses remains a challenge. These viruses usually cause overlapping clinical syndromes, making it difficult to assign viral aetiology based on the clinical presentations of patients [2]. Moreover, laboratory tests necessary for case confirmation are not always conducted in clinical settings owing to limited laboratory capacity [3]. Previous studies have used several statistical methods to quantify the morbidity and mortality burden associated with influenza and respiratory syncytial viruses (RSV) [4]. These methods first established a baseline level with the assumption of no virus circulation, and then defined the excess hospitalization or mortality as the difference between the observed and baseline. However, few of these methods were able to separately determine the burden attributable to different respiratory viruses and even fewer studies have assessed the burden of respiratory viruses other than influenza and RSV. One commonly used method, Poisson regression modeling, allows simultaneous assessment of co-circulating viruses and has become increasingly popular recently. But our previous study showed that the point estimates derived by the classical maximum likelihood method for respiratory viruses other than influenza were unrealistically small and even negative [5]. The challenge lies in resolving the overlapping peaks of these co-circulating viruses, and also in adjusting for the confounding effects of other seasonal factors such as temperature or humidity [6]. An alternative estimation method, Bayesian inference, could be used as it has the advantage of incorporating the prior knowledge on parameter distributions [7]. Another unsolved problem in disease burden studies is the choice of virus proxy variables. The numbers or proportions of specimens positive for different viruses in all specimens tested have been widely used in previous studies [8], [9]. Other less frequently used proxies include influenza-like illness rates multiplied by laboratory-test positive proportions (ILI×LAB) [10]. Although virus attack rates could be different across age groups due to the heterogeneity in prior immunity and exposure risks [11]–[13], no studies have hitherto integrated age-specific virus data into the models, largely due to the lack of such data in most regions. In this study we evaluated the performance of various combinations of model assumption, virus proxy variables and inference methods, in estimating excess hospitalization attributable to several co-circulating respiratory viruses. The estimates have been validated by comparison with observed rates of laboratory confirmed paediatric hospitalization rates for acute respiratory infections obtained from a population based study.

## Methods

### Data source

Hospital admission records of the two major public hospitals on the Hong Kong Island (Queen Mary Hospital (QMH) and Pamela Youde Nethersole Eastern Hospital (PYNEH) were obtained from the Hong Kong Hospital Authority during the study period of October 2003–September 2010. We compiled weekly numbers of hospital admissions with any listed discharge diagnosis of acute respiratory diseases (ARD) for the age groups of <1, 1–5 and 6–17 years, according to the International Classification of Diseases (9th Revision, ICD9) codes 460–466 or 480–487. Age specific virology data were obtained from the Microbiology Laboratory of QMH, which provides virology diagnostic services for both QMH and PYNEH, for influenza A (seasonal subtypes H3N2, sH1N1 and pandemic strain pH1N1), influenza B, respiratory syncytial virus (RSV), adenovirus and parainfluenza virus types 1–3. This laboratory tested a total of 80 611 specimens collected from both QMH and PYNEH during the study period, by using direct immunofluorescence tests (IF) and viral culture. Reverse transcription polymerase chain reaction (RT-PCR) was only routinely carried out during the 2009 pandemic [14]. Meteorological data were obtained from the Hong Kong Observatory.

### Poisson model

Poisson models were first fitted to the age-stratified weekly admission numbers of acute respiratory diseases. A typical form of this model is
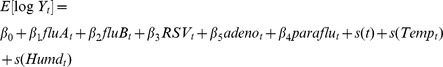


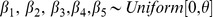
(Model 1)where *Y_t_* denotes the numbers of age-specific hospital admissions at week *t (t = 1,2,…,366)*, and follows a Poisson distribution with mean *μ_t_* and variance *φμ_t_*. Here *φ* is an over-dispersion factor to adjust for the unequal mean and variance [15]. *fluA_t_*, *fluB_t_*, *RSV_t_*, *adeno_t_* and *paraflu_t_* denote the age-specific weekly counts of specimens positive for influenza A and B, RSV, adenovirus or parainfluenza viruses, respectively. 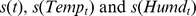 are the natural spline functions of time, weekly average temperature and relative humidity, respectively. Five degrees of freedom per year were used for the seasonal trend and two degrees of freedom for temperature and relative humidity. We used a Bayesian inference process based on Gibbs sampling (BUGS) [16] to estimate the parameters. A variety of Bayesian approaches have been widely applied to calculate the genetic distance in phylogenetic analysis [17] and to describe the transmission dynamics of influenza viruses [18]. By incorporating prior knowledge on the distribution of parameter with available data, the Bayesian inference method could provide a posterior distribution closer to the true underlying distribution [19]. Due to the known adverse effects of the viruses on hospital admissions, we assumed that the parameter of virus proxy variable followed a non-negative distribution. Therefore the coefficients of these variables *β_1_*, *β_2_*, *β_3_*, *β_4_* and *β_5_* were estimated by a Bayesian process, under the distribution assumption of *Uniform[0,θ]*. The posterior distribution of each covariate parameter was estimated by repeating a Monte Carlo Markov Chain simulation for 50,000 iterations with 25,000 burn-in iterations. Based on our previous findings [20], the starting point of *θ* was set to 10, to cover the range of excess risk from 0–20% associated with 10% increase in virus proxies.

In addition to age-specific positive counts, we tried different combinations of virus proxies with the Bayesian inference method on virus coefficients: age-specific proportions of positive specimens (Model 2), all-ages proportions (Model 3) or all-ages influenza-like illness rates multiplied by all-ages proportions (ILI×LAB, model 4). Besides the commonly adopted log linear Poisson regression models that assumed multiplicative effects of viruses, we also tried linear Gaussian models that assumed additive effects of influenza (Model 5) [10], [21]. To compare the Bayesian approach with our previous models based on classical likelihood estimation, we fitted the classical log linear Poisson models with the proxies of age-specific counts (Model 6), age-specific proportions (Model 7) and all-ages proportions (Model 8).

### Model validation

Baseline hospitalization for influenza A subtype H3N2 was first calculated from the model as the expected weekly numbers of admissions when the H3N2 proxy variable was set to zero and all the other variables were kept as the observed values. Excess hospitalization attributable to H3N2 was defined as the sum of difference between the observed and baseline hospitalization [22]. Similar calculation was repeated for other subtypes of influenza A, influenza B, RSV, adenovirus and parainfluenza, respectively. Annual excess rate of hospitalization was separately calculated for each year, by dividing the annual total number of excess hospitalization by the mid-year age-specific population in the Hong Kong Island obtained from the year 2006 census.

Annual excess rates estimated by these statistical methods were then compared with the directly observed admission rates for a population based systematic sample of laboratory confirmed cases of respiratory virus infections, who were admitted into the QMH and PYNEH with any listed diagnosis of ARD during the same period. The details of data collection for the directly observed virologically confirmed hospitalization rates have been described elsewhere [23]. Briefly, nasopharyngeal aspirates from patients who were younger than 18 years and admitted with symptoms of acute respiratory infection on one chosen day (Wednesday or Thursday) of each week, were all tested for five respiratory viruses by IF. Since these two hospitals provide acute paediatric hospital services for approximately 70% of the population in Hong Kong Island, we could estimate the population based age-specific hospitalization rates from this cohort. We calculated the mean of absolute percentage difference between the annual age-specific estimates and corresponding virologically confirmed observed hospitalization rates, and chose the most optimal model as that with the smallest mean difference. We also assessed the lag effects of these viruses by replacing the virus proxy variables with the proxies at the weeks up to three weeks before the current (lag1, 2 and 3), to take into account of the potential delay between the virus infection and hospital admissions. For simplicity, the same lag was used for all the virus proxies in the model. In order to assess whether our method could differentiate the impacts of viruses during the interpandemic and pandemic periods, we calculated the excess rates separately for the 2009 H1N1 influenza pandemic period of May 2009 to August 2010, and for the preceding interpandemic period of October 2003 to April 2009. All the analysis was performed by the statistical packages R (version 2.5.1) and WinBUGS (version 1.4.3).

Ethical approval was obtained from the Institutional Review Board of the University of Hong Kong/Hospital Authority Hong Kong West Cluster (UW 11-264). Informed consent was not obtained because patient records were anonymized and de-identified prior to analysis.

## Results

The mean absolute percentage difference between the annual age-specific rates of excess hospitalization derived from different models and the corresponding observed rates is shown in Table 1. In the models using the same virus proxies, the estimates from the models using the Bayesian inference showed smaller deviations from the observed rates than the classical likelihood estimates, particularly for RSV, parainfluenza and adenovirus. In the models using the Bayesian inference, compared to the other virus proxies, age-specific counts provided the estimates with smaller deviation from the true observed rates for most viruses (Table 1). The log-link models (Model 1) offered the estimates closer to the observed rates than the identity-link models (Model 5), with the exception of parainfluenza. Overall, the log-link Poisson models using the Bayesian inference and the proxies of age-specific counts (Model 1) provided the most reliable estimates for the excess hospitalization associated with influenza A and B, RSV, parainfluenza and adenoviruses. Therefore we chose this model as the final one and presented the estimates from this model in the rest part of this paper. The lag effects up to three weeks were separately assessed by replacing the age-specific positive counts virus at the current week (lag 0) with those at one to three weeks before (lag 1–3). These models with different lag week consistently provided the estimates more deviant from the observed rates, compared to the proxy variables at the current week (Table 2).

**Table 1:** Mean absolute percentage difference between annual age-specific excess hospitalization rates and annual hospitalization rates of laboratory confirmed infections in a pediatric cohort.

	Model 1	Model 2	Model 3	Model 4	Model 5	Model 6	Model 7	Model 8
Link function	Log	Log	Log	Log	Linear	Log	Log	Log
Inference	Bayesian	Bayesian	Bayesian	Bayesian	Bayesian	Classical	Classical	Classical
Prior	Uniform	Uniform	Uniform	Uniform	Uniform	NA	NA	NA
Virus proxies	Age-specific count	Age-specific proportion	All-ages proportion	ILI×LAB	Age-specific count	Age-specific count	Age-specific proportion	All-ages proportion
Influenza A								
sH1N1	38.4	43.3	40.6	33.9	46.6	41.0	77.4	44.0
H3N2	38.8	28.4	71.4	33.9	48.0	38.7	27.7	93.8
pH1N1	37.8	74.5	47.1	43.6	41.5	37.0	48.7	49.7
Influenza B	54.3	43.2	57.3	58.7	64.9	57.0	53.9	74.3
RSV	39.1	46.1	45.3	55.7	43.6	39.4	78.3	279.4
Parainfluenza	53.8	61.6	71.1	81.4	49.2	53.6	88.1	215.3
Adenovirus	63.4	79.1	68.8	65.3	68.3	68.2	108.9	145.0

Abbreviations: RSV, respiratory syncytial virus; NA, not available.

**Table 2:** Mean absolute percentage difference of excess hospitalization rates from annual hospitalization rates of laboratory confirmed infections in a pediatric cohort.

Lag weeks	Lag 1	Lag 2	Lag 3
Influenza A			
sH1N1	52.5	70.9	81.5
H3N2	44.5	62.5	70.0
pH1N1	57.8	68.5	78.5
Influenza B	41.7	62.7	72.5
RSV	62.2	102.6	77.6
Parainfluenza	65.2	94.9	86.4
Adenovirus	65.4	71.6	62.8

Excess rates were estimated from the log-linear Poisson model using a Bayesian approach with the virus proxies of age-specific positive counts at the different lag weeks.

**Figure 1:**
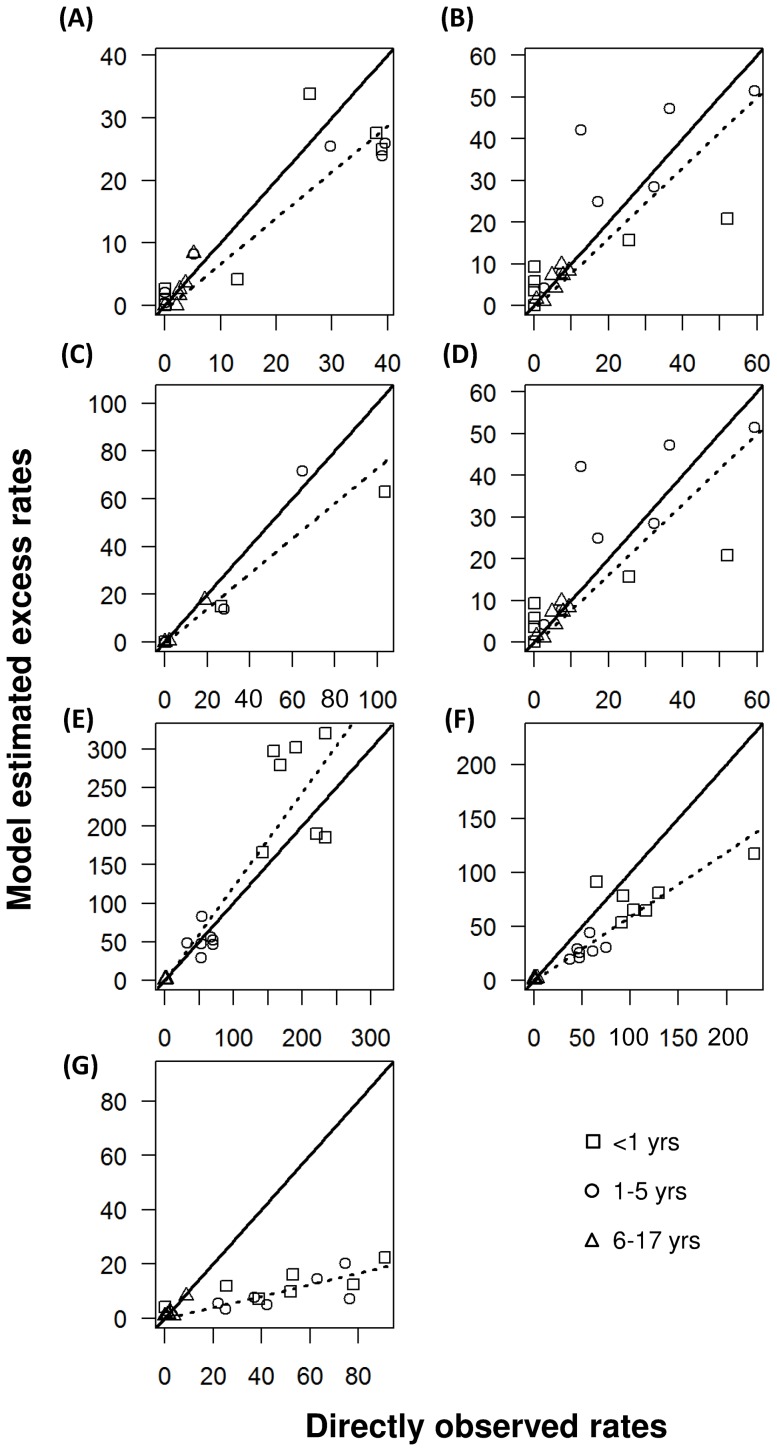
Comparison of annual excess hospitalization rates per 10,000 population and directly observed rates during each of the seven seasons, for (A) sH1N1, (B) H3N2, (C) pH1N1, (D) influenza B, (E) respiratory syncytial virus (RSV), (F) parainfluenza and (G) adenovirus. Excess hospitalization rates were derived from the WinBUGS models with age-specific counts as virus proxy.

Annual excess rates of hospitalization were slightly lower than the directly observed rates for influenza A subtypes sH1N1, H3N2, pH1N1 and influenza B in all the age groups, without any pattern of consistent under- or over-estimation observed in any of these age groups (Figure 1). For RSV, excess rates tended to be higher than the observed hospitalization rates, particularly for the <1 age groups. Most of the estimates for parainfluenza were smaller than the observed rates. The greatest deviation from the observed rates was found in adenovirus.

Compared to the interpandemic period, the 2009 H1N1 pandemic was associated with an obvious increase in the observed rates of laboratory confirmed cases for RSV, but a decrease in other viruses (Table 3). Overall the model provided the estimates similar to the directly observed rates of all the viruses under study during the pandemic period, except slight overestimation in H3N2 and influenza B, and underestimation in adenovirus. The model performance was comparable between the interpandemic and pandemic periods for all the viruses.

**Table 3:** Comparison of weekly directly observed rates (per 100,000 population) and excess rates of hospitalization associated with influenza estimated by the Bayesian approach, during the interpandemic period (4 January 2004–25 April 2009) and pandemic period (26 April 2009–14 August 2010).

Virus/Age group	Interpandmic		Pandemic	
	Directly observed rates	Excess rates (95% CI)	Directly observed rates	Excess rates (95% CI)
sH1N1				
<1	4.2	3.2 (0.3, 6.6)	0.0	0.4 (0.0, 1.0)
1–5	3.9	2.9 (1.0, 4.6)	0.7	0.8 (0.3, 1.3)
6–17	0.6	0.6 (0.3, 0.9)	0.1	0.1 (0.0, 0.1)
H3N2				
<1	8.9	12.0 (6.9, 17.3)	5.7	8.1 (4.6, 11.6)
1–5	6.2	9.0 (7.0, 11.0)	5.1	6.4 (4.9, 8.0)
6–17	0.7	0.9 (0.6, 1.2)	0.3	0.9 (0.6, 1.3)
pH1N1				
<1	na	na	17.2	11.3 (1.2, 21.9)
1–5	na	na	12.4	12.2 (8.2, 16.3)
6–17	na	na	2.9	2.6 (2.0, 3.2)
Influenza B				
<1	2.8	1.9 (0.1, 5.1)	0.0	0.8 (0.0, 2.2)
1–5	5.6	5.9 (4.0, 8.0)	0.7	5.2 (3.4, 7.0)
6–17	1.0	1.1 (0.8, 1.4)	1.0	1.1 (0.8, 1.6)
RSV				
<1	36.5	48.1 (38.7, 57.0)	45.8	48.3 (39.3, 57.9)
1–5	10.6	9.0 (5.1, 12.6)	12.7	12.4 (7.0, 17.2)
6–17	0.1	0.3 (0.0, 0.7)	0.2	0.4 (0.0, 1.1)
Parainfluenza				
<1	24.8	15.9 (8.0, 23.7)	13.3	11.6 (5.7, 17.6)
1–5	9.9	5.0 (1.8, 8.0)	10.9	6.3 (2.2, 10.3)
6–17	0.1	0.2 (0.1, 0.4)	0.3	0.4 (0.1, 0.8)
Adenovirus				
<1	10.3	2.4 (0.1, 5.4)	5.7	2.2 (0.1, 5.1)
1–5	9.6	1.7 (0.1, 4.3)	5.5	1.0 (0.0, 2.6)
6–17	0.6	0.5 (0.2, 0.9)	0.1	0.2 (0.1, 0.3)

Abbreviations: RSV, respiratory syncytial virus; NA, not available.

## Discussion

**Figure 2:**
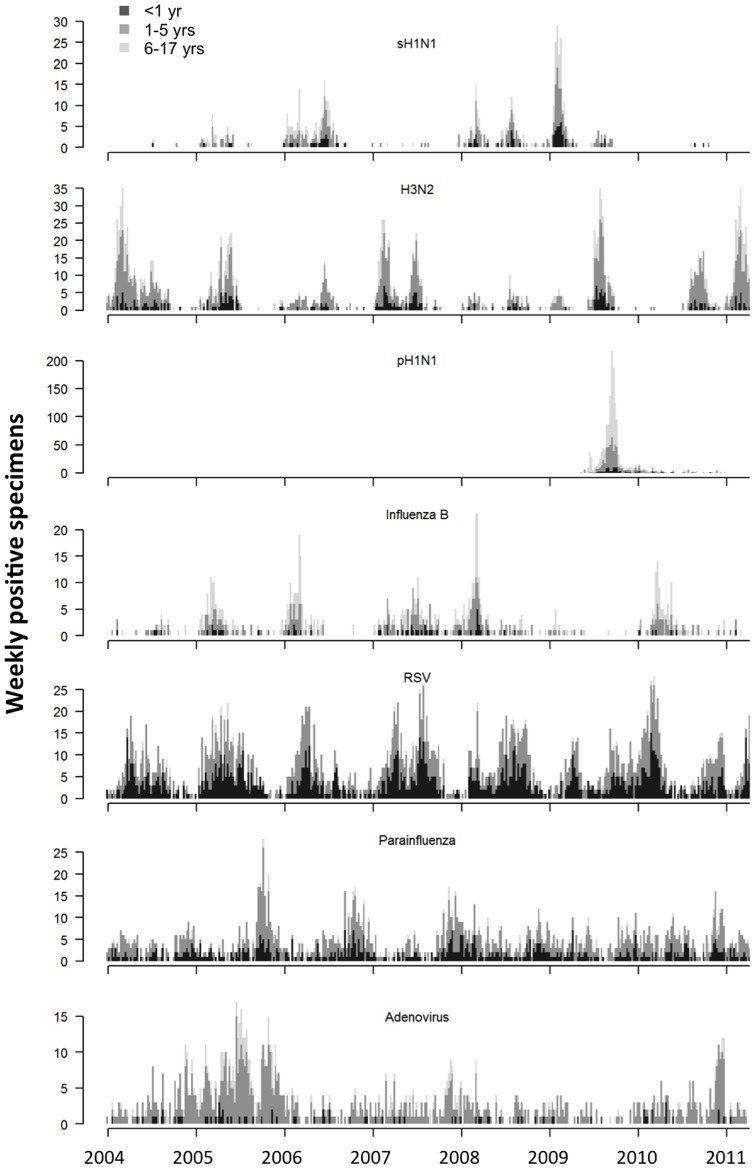
Weekly numbers of specimens positive for influenza A or B, RSV, parainfluenza and adenovirus in the age groups of <1, 1–5 and 6–17 years.

Time series models have widely adopted by recent studies to estimate the disease burden of influenza and RSV [24], [25]. In this study we compared the Bayesian inference method with the classical likelihood estimation, in terms of obtaining more reliable estimates for the disease burden of co-circulating viruses including influenza, RSV, parainfluenza and adenovirus. Under the assumption of positive association between respiratory virus activity and hospitalization, the Bayesian inference method successfully separated the individual effects of multiple respiratory viruses, which the previous models have not or only partially achieved [5], [26]. With the exception of adenovirus, the model estimates closely matched the true hospitalization rates across different age groups that were observed in a pediatric cohort under a systematic surveillance for respiratory virus infections. We speculated that underestimation in adenovirus was probably due to its less clear seasonal pattern and relatively lower positive isolation rate compared to the other viruses (Figure 2). Nevertheless, the models overall offered the satisfactory estimates which were within the close range of true hospitalization rates without exaggeration.

**Figure 3:**
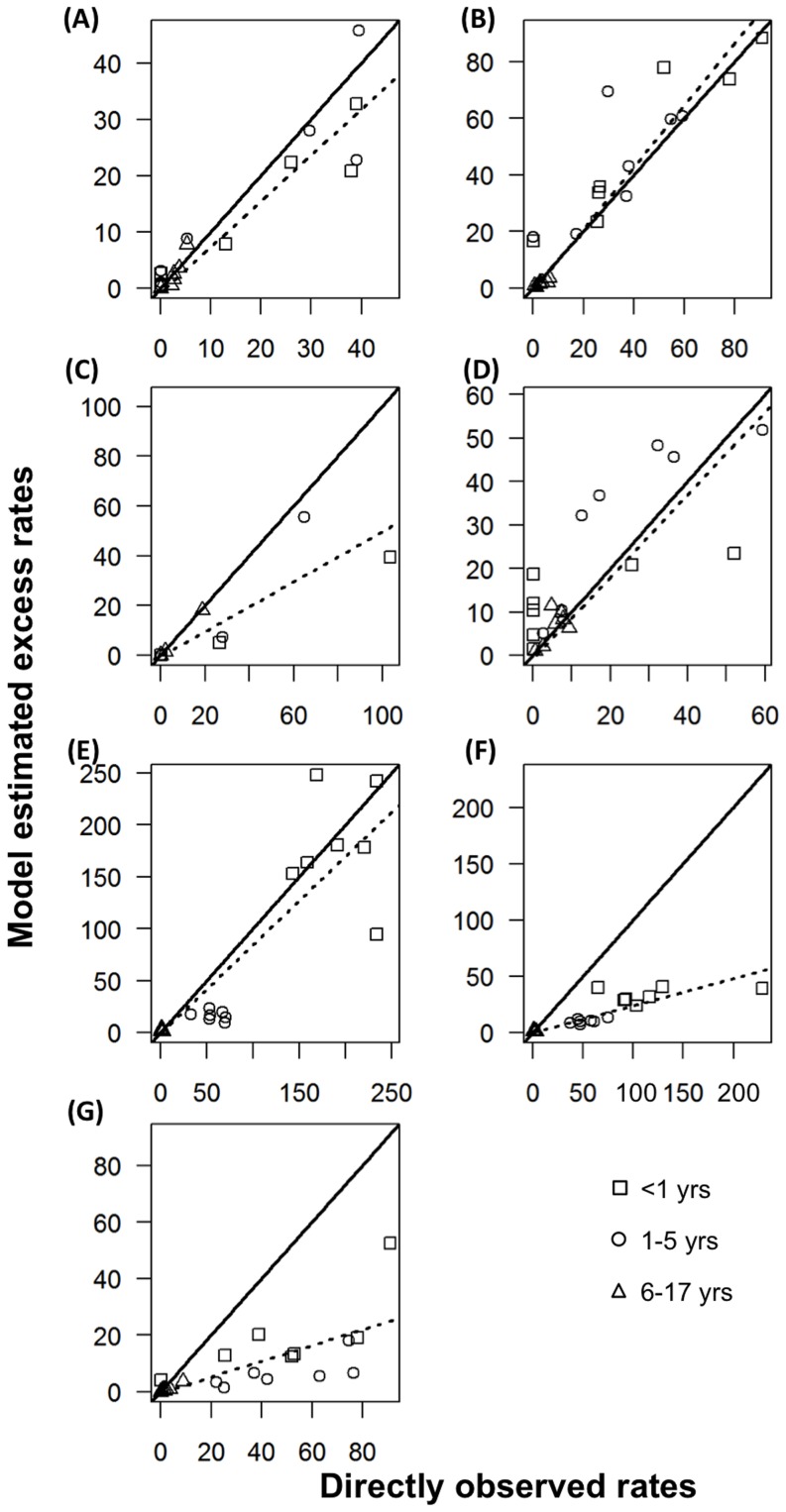
Comparison of annual excess hospitalization rates per 10,000 population and directly observed rates during each of the seven seasons, for (A) sH1N1, (B) H3N2, (C) pH1N1, (D) influenza B, (E) respiratory syncytial virus (RSV), (F) parainfluenza and (G) adenovirus. Excess hospitalization rates were derived from the Poisson models with the virus proxies of influenza-like illness rates multiplied by virus proportions (ILI×LAB).

Taking the advantage of long standing virology data with linked age information in Hong Kong, this study for the first time added the age-specific virology data as proxy in the time series models for disease burden studies. We found that age-specific counts showed the best performance among all the proxies when combined with either the Bayesian or classical likelihood inference methods. In previous studies, we used all-ages proportion as proxy because it took into account the temporal variations in total numbers of specimens collected. However, this might not be the case for age-specific virology data, as relatively small numbers of total specimens tested in some age groups could have introduced spurious peaks in age-specific proportions. We also evaluated the performance of ILI×LAB proxy, which was found more closely correlated with the true incidence of influenza during the interpandemic or pandemic period [21], [27]. We found this proxy provides the estimates closer to the observed rates than age-specific and all-ages proportions, but slightly worse than the proxy of age-specific counts in most viruses (Table 1 and Figure 3). Taken together, age-specific counts shall be recommended as proxy variables if such data are available. If age information is unavailable, ILI×LAB is probably the proxy that shall be considered.

The 2009 H1N1 pandemic was characterized with dramatically increased attack rates among children and young adults, but the severity of pandemic infections was comparable to the seasonal virus strains [28], [29]. ARD admission rates in our pediatric cohort increased by a proportion ranging from 7% to 170% during the pandemic (Table 1), and many other studies also reported a similar magnitude of increase [30]–[33]. However, the admissions due to non-influenza infections decreased in the pandemic, except RSV. Our model estimates were able to capture this trend, showing the same change directions as the observed rates. However, large deviations were also observed in some age-virus categories, such as influenza B in the <1 and 1–5 age groups. Further studies are warranted to fine tune our modeling approach in order to derive reliable estimates for different periods.

It has been widely accepted that Poisson distribution is appropriate to fit the low-frequency count data, but the log-link function commonly adopted in Poisson models has been criticized for its assumption of exponential increase in health outcomes along with one unit increase in virus proxies [8], [34]. Some of recent studies switched to a more “reasonable” assumption of linear relation by adopting an identity-link function in Poisson models [35], [36]. In this study we found that the log-link function yielded the estimates slightly closer to the true incidence of influenza hospitalizations than the identity-link. However, the key assumption on the association of virus proxies and health outcomes in Poisson models still remain to be proved. Further evidence on the mechanism of influenza transmission and pathogenicity in human community could probably help resolve this problem.

Our study has potential limitations. First, the Bayesian estimates are sensitive to the prior distributions and the prior assumption of nonnegative coefficient for virus proxy variables needs to be carefully justified. Since our virology data were obtained from the laboratory surveillance based on hospitalized inpatients, it is reasonable to assume that these virology data were positively associated with the increase of hospital admissions with viral respiratory infections. However, overestimation might exist if the assumption of prior distribution is not well justified, and caution needs to be taken when extending this approach to estimate the excess mortality of other respiratory viruses, as most viruses other than influenza cause only mild symptoms that might not necessarily lead to death [37]. Second, age-specific virus data requires long standing and intensive virology surveillance for multiple respiratory viruses, but such surveillance networks may not be available for influenza in many countries. Nevertheless, the importance of simultaneous assessment on other respiratory viruses, particularly RSV, has started to be recognized [26], [38]. So we can expect these data will become available in more and more countries in the near future. Third, we only estimated the excess hospitalization of five respiratory viruses due to limited virology data. There are many other respiratory viruses (e.g. rhinovirus) and bacteria (e.g. *Streptococcus pneumonia*) also contribute greatly to ARD hospitalization in children, although the clinical significance of detection of some of these (e.g. rhinovirus) remains unclear. Further studies are needed to assess whether addition of more virology data could alter the performance of models.

In conclusion, age-specific counts of positive specimens are probably the best proxies for virus activity and should be used in the disease burden models if such data are available. In the absence of age-specific data, the Bayesian inference proposed in this study is superior to the classical likelihood inference method, as the former provides more reliable estimates on excess hospitalization respectively associated with multiple respiratory viruses.

## References

[1] Williams BG, Gouws E, Boschi-Pinto C, Bryce J, Dye C (2002) Estimates of world-wide distribution of child deaths from acute respiratory infections. Lancet Infect Dis 2: 25–32. 11892493 10.1016/s1473-3099(01)00170-0

[2] Monto AS, Gravenstein S, Elliott M, Colopy M, Schweinle J (2000) Clinical signs and symptoms predicting influenza infection. ArchInternMed 160: 3243–3247. 10.1001/archinte.160.21.3243 11088084

[3] Fleming DM, Elliot AJ, Cross KW (2007) Morbidity profiles of patients consulting during influenza and respiratory syncytial virus active periods. EpidemiolInfect 135: 1099–1108. 10.1017/S0950268807007881 PMC2870675 17291381

[4] Thompson WW, Weintraub E, Dhankhar P, Cheng PY, Brammer L, et al (2009) Estimates of US influenza-associated deaths made using four different methods. InfluenzaOther RespiViruses 3: 37–49. 10.1111/j.1750-2659.2009.00073.x PMC4986622 19453440

[5] Yang L, Chiu SS, Chan KP, Chan KH, Wong WH, et al (2011) Validation of statistical models for estimating hospitalization associated with influenza and other respiratory viruses. PLoS One 6: e17882. 21412433 10.1371/journal.pone.0017882 PMC3055891

[6] Dowell SF (2001) Seasonal variation in host susceptibility and cycles of certain infectious diseases. EmergInfect Dis 7: 369–374. 10.3201/eid0703.010301 PMC2631809 11384511

[7] Dellaportas P, Smith AFM (1993) Bayesian Inference for Generalized Linear and Proportional Hazards Models via Gibbs Sampling. Journal of the Royal Statistical Society Series C (Applied Statistics) 42: 443–459.

[8] Thompson WW, Shay DK, Weintraub E, Brammer L, Cox N, et al (2003) Mortality associated with influenza and respiratory syncytial virus in the United States. JAMA 289: 179–186. 12517228 10.1001/jama.289.2.179

[9] Wong CM, Chan KP, Hedley AJ, Peiris JSM (2004) Influenza-associated mortality in Hong Kong. Clin Infect Dis 39: 1611–1617. 15578360 10.1086/425315

[10] Wu P, Goldstein E, Ho LM, Yang L, Nishiura H, et al (2012) Excess mortality associated with influenza A and B virus in Hong Kong, 1998–2009. Journal of Infectious Diseases 206: 1862–1871. 23045622 10.1093/infdis/jis628 PMC3502382

[11] Hui SL, Chu LW, Peiris JSM, Chan KH, Chu D, et al (2006) Immune response to influenza vaccination in community-dwelling Chinese elderly persons. Vaccine 24: 5371–5380. 16713661 10.1016/j.vaccine.2006.04.032

[12] Munoz FM (2003) Influenza virus infection in infancy and early childhood. Paediatric Respiratory Reviews 4: 99–104. 12758046

[13] Olson DR, Heffernan RT, Paladini M, Konty K, Weiss D, et al (2007) Monitoring the impact of influenza by age: emergency department fever and respiratory complaint surveillance in New York City. PLoS Med 4: e247. 17683196 10.1371/journal.pmed.0040247 PMC1939858

[14] Chan KH, Peiris JS, Lim W, Nicholls JM, Chiu SS (2008) Comparison of nasopharyngeal flocked swabs and aspirates for rapid diagnosis of respiratory viruses in children. J Clin Virol 42: 65–69. 18242124 10.1016/j.jcv.2007.12.003

[15] Hastie TJ, Tibshirani RJ (1990) Generalized additive models. London: Chapman and Hall. 10.1177/096228029500400302 8548102

[16] Lunn DJ, Thomas A, Best N, Spiegelhalter D (2000) WinBUGS - A Bayesian modelling framework: Concepts, structure, and extensibility. Statistics and Computing 10: 325–337.

[17] Bahl J, Nelson MI, Chan KH, Chen R, Vijaykrishna D, et al (2011) Temporally structured metapopulation dynamics and persistence of influenza A H3N2 virus in humans. Proc Natl Acad Sci U S A 108: 19359–19364. 22084096 10.1073/pnas.1109314108 PMC3228450

[18] Birrell PJ, Ketsetzis G, Gay NJ, Cooper BS, Presanis AM, et al (2011) Bayesian modeling to unmask and predict influenza A/H1N1pdm dynamics in London. Proceedings of the National Academy of Sciences 108: 18238–18243. 10.1073/pnas.1103002108 PMC3215054 22042838

[19] Christensen R, Johnson W, Branscum A, Hanson TE (2011) Bayesian ideas and data analysis. US: CRC Press.

[20] Yang L, Chen PY, He JF, Chan KP, Ou CQ, et al (2011) Effect modification of environmental factors on influenza-associated mortality: a time-series study in two Chinese cities. BMC Infect Dis 11: 342. 22168284 10.1186/1471-2334-11-342 PMC3265445

[21] Goldstein E, Viboud C, Charu V, Lipsitch M (2012) Improving the Estimation of Influenza-Related Mortality Over a Seasonal Baseline. Epidemiology 23: 829–838. 22992574 10.1097/EDE.0b013e31826c2dda PMC3516362

[22] Wong CM, Yang L, Chan KP, Leung GM, Chan KH, et al (2006) Influenza-associated hospitalization in a subtropical city. PLoS Med 3: e121. 16515368 10.1371/journal.pmed.0030121 PMC1391978

[23] Chiu SS, Chan KH, Chen H, Young BW, Lim W, et al (2009) Virologically confirmed population-based burden of hospitalization caused by influenza A and B among children in Hong Kong. Clin Infect Dis 49: 1016–1021. 19722912 10.1086/605570

[24] Charu V, Simonsen L, Lustig R, Steiner C, Viboud C (2013) Mortality burden of the 2009–10 influenza pandemic in the United States: improving the timeliness of influenza severity estimates using inpatient mortality records. Influenza Other Respi Viruses 10.1111/irv.12096 PMC3674131 23419002

[25] Redlberger-Fritz M, Aberle JH, Popow-Kraupp T, Kundi M (2012) Attributable deaths due to influenza: a comparative study of seasonal and pandemic influenza. Eur J Epidemiol 27: 567–575. 22678614 10.1007/s10654-012-9701-y

[26] van Asten L, van den Wijngaard C, van Pelt W, van de Kassteele J, Meijer A, et al (2012) Mortality Attributable to 9 Common Infections: Significant effect of influenza A, RSV, influenza B, norovirus and parainfluenza in the elderly. J Infect Dis 206: 628–639. 22723641 10.1093/infdis/jis415

[27] Wong JY, Wu P, Nishiura H, Goldstein E, Lau EH, et al (2013) Infection Fatality Risk of the Pandemic A(H1N1)2009 Virus in Hong Kong. Am J Epidemiol 177: 834–840. 23459950 10.1093/aje/kws314 PMC3658096

[28] Wu JT, Ho A, Ma ES, Lee CK, Chu DK, et al (2011) Estimating Infection Attack Rates and Severity in Real Time during an Influenza Pandemic: Analysis of Serial Cross-Sectional Serologic Surveillance Data. PLoSMed 8: e1001103. 10.1371/journal.pmed.1001103 PMC3186812 21990967

[29] Belongia EA, Irving SA, Waring SC, Coleman LA, Meece JK, et al (2010) Clinical characteristics and 30-day outcomes for influenza A 2009 (H1N1), 2008–2009 (H1N1), and 2007–2008 (H3N2) infections. JAMA 304: 1091–1098. 20823435 10.1001/jama.2010.1277

[30] Hernandez JE, Grainger J, Simonsen L, Collis P, Edelman L, et al (2012) Impact of the 2009/2010 influenza A (H1N1) pandemic on trends in influenza hospitalization, diagnostic testing, and treatment. Influenza Other Respi Viruses 6: 305–308. 10.1111/j.1750-2659.2011.00303.x PMC5779814 22085222

[31] Engelhard D, Bromberg M, Averbuch D, Tenenbaum A, Goldmann D, et al (2011) Increased extent of and risk factors for pandemic (H1N1) 2009 and seasonal influenza among children, Israel. Emerg Infect Dis 17: 1740–1743. 21888809 10.3201/eid1709.102022 PMC3322075

[32] Karageorgopoulos DE, Vouloumanou EK, Korbila IP, Kapaskelis A, Falagas ME (2011) Age distribution of cases of 2009 (H1N1) pandemic influenza in comparison with seasonal influenza. PLoS ONE 6: e21690. 21747947 10.1371/journal.pone.0021690 PMC3128617

[33] Song X, DeBiasi RL, Campos JM, Fagbuyi DB, Jacobs BR, et al (2012) Comparison of pandemic and seasonal influenza A infections in pediatric patients: were they different? Influenza Other Respi Viruses 6: 25–27. 10.1111/j.1750-2659.2011.00258.x PMC4941554 21668668

[34] Thompson WW, Ridenhour BL, Barile JP, Shay DK (2012) Time-series analyses of count data to estimate the burden of seasonal infectious diseases. Epidemiology 23: 839–842. 23038110 10.1097/EDE.0b013e31826cc1df

[35] Newall AT, Viboud C, Wood JG (2010) Influenza-attributable mortality in Australians aged more than 50 years: a comparison of different modelling approaches. Epidemiol Infect 138: 836–842. 19941685 10.1017/S095026880999118X

[36] Lemaitre M, Carrat F, Rey G, Miller M, Simonsen L, et al (2012) Mortality burden of the 2009 A/H1N1 influenza pandemic in France: comparison to seasonal influenza and the A/H3N2 pandemic. PLoS One 7: e45051. 23028756 10.1371/journal.pone.0045051 PMC3447811

[37] Foppa IM, Hossain MM (2008) Revised estimates of influenza-associated excess mortality, United States, 1995 through 2005. Emerg Themes Epidemiol 5: 26. 19116016 10.1186/1742-7622-5-26 PMC2628891

[38] Zhou H, Thompson WW, Viboud CG, Ringholz CM, Cheng PY, et al (2012) Hospitalizations Associated With Influenza and Respiratory Syncytial Virus in the United States, 1993–2008. Clin Infect Dis 54: 1427–1436. 22495079 10.1093/cid/cis211 PMC3334364

